# Genomic Alterations of the Infectious Bronchitis Virus (IBV) Strain of the GI-23 Lineage Induced by Passages in Chickens and Quails

**DOI:** 10.3390/ijms26094200

**Published:** 2025-04-28

**Authors:** Katarzyna Domanska-Blicharz, Joanna Sajewicz-Krukowska, Anna Lisowska, Justyna Opolska, Karolina Tarasiuk, Kamila Dziadek

**Affiliations:** Department of Virology and Viral Animal Diseases, National Veterinary Research Institute, al. Partyzantów 57, 24-100 Puławy, Poland; sajewicz@piwet.pulawy.pl (J.S.-K.); lisowska@piwet.pulawy.pl (A.L.); opolska@piwet.pulawy.pl (J.O.); tarasiuk@piwet.pulawy.pl (K.T.); kamila.dziadek@piwet.pulawy.pl (K.D.)

**Keywords:** infectious bronchitis virus, IBV, GI-23, Var2, virus evolution, chickens, quails

## Abstract

Infectious bronchitis virus (IBV) of the GI-23 lineage, which first emerged in the Middle East in the late 1990s, has since spread worldwide. The factors driving its expansion, whether human involvement, wild bird migration, or the virus’s biological traits, are still unclear. This study aimed to trace the genome evolution of GI-23 IBV in chickens and its adaptability to quails, which are susceptible to both gamma- and deltacoronaviruses. Thirty specific-pathogen-free (SPF) birds, aged between two and three weeks, were used. Initially, three birds were inoculated with the G052/2016 IBV via the oculo-nasal route. On the third day post-infection (dpi), oropharyngeal swabs were collected from the whole group, pooled, and subsequently used to infect three next birds. This process was repeated nine more times during consecutive IBV passages (P-I–P-X), and eventually, virus sequencing was performed using Next-Generation Sequencing (NGS). The obtained results showed that quails were not susceptible to the IBV GI-23 lineage, as the virus RNA was detected in low amounts only during the first passage (QP-I) with no further detections in later rounds of IBV passaging. In chickens, only mild diarrhea symptoms appeared in a few individuals. The NGS analysis identified sixty-two single nucleotide variants (SNVs), thirty of which caused amino acid changes, twenty-eight were synonymous, and one SNV introduced a stop codon. Three SNVs were found in untranslated regions. However, none of these SNVs lasted beyond seven passages, with forty-four being unique SNVs. The Shannon entropy values measured during passages varied for *pol1a*, *pol1b*, *S*, *5a*, *5b*, and *N* genes, with overall genome complexity peaking at CP-VI and CP-X. The highest complexity was observed in the *pol1a* (CP-X) and *S* genes (CP-IV, CP-VI, CP-VIII, and CP-X). Along with the *S* gene that was under positive selection, eight codons in *pol1a* were also positively selected. These findings suggest that even in an adapted host, IBV variability does not stabilize without immune pressure, indicating continuous molecular changes within its genome.

## 1. Introduction

Infectious bronchitis (IB), initially described in 1931 as a respiratory disease of chickens, was later found to affect other systems as well, including the reproductive, gastrointestinal, and urinary systems [1,2,3]. This multisystemic nature of the disease is primarily attributed to the high variability of the infectious bronchitis virus (IBV). Both IBV and its variant, IBV Ind-Tn92-03 (also known as AvCoV 9203), are the causative agents of IB and represent distinct species within the Gammacoronavirus genus: *Gammacoronavirus galli* and *Gammacoronavirus pulli*, respectively. These two species, along with *Gammacoronavirus anatis*, belong to the subgenus *Igacovirus* [4]. Similar to other coronaviruses, IBV has a large, unsegmented, single-stranded, positive-sense RNA genome of about 27 kb. The 5′ end of the genome contains two overlapping open reading frames (ORF1a and ORF1b), which encode two large polyproteins. These polyproteins are cleaved into 15 non-structural proteins that form the viral replicase machinery. The remaining third of the genome consists of structural and accessory genes, arranged in the following order: spike (S), accessory genes *3a* and *3b*, envelope (E), membrane (M), accessory genes *4b*, *5a*, and *5b*, and nucleocapsid (N) [5]. IBV replication is driven by both low-fidelity RNA-dependent RNA polymerase (RdRP) and high-fidelity proofreading enzyme, i.e., exoribonuclease (ExoN). This, combined with the discontinuous nature of coronavirus transcription, results in a high mutation rate in progeny viruses, including point mutations, insertions, deletions, and recombinations [6,7]. Although most of these altered progeny viruses do not survive, advantageous mutations can result in viruses with modified pathogenicity, different disease manifestations, and even the ability to switch hosts, as seen with turkey coronaviruses [8,9]. IBV exhibits considerable genetic diversity, with a variety of different genotypes and antigenic types identified. Genotypically, IBV is classified into eight genotypes, denoted by Roman numerals, with each genotype containing multiple lineages (designated with Arabic numerals). Additionally, unique variants that are not assigned to any genotype have been identified [10].

In Poland, several IBV genotypes and lineages, such as Mass (GI-1), 793B (GI-13), QX (GI-19), and Var2 (GI-23), but also D1466 (GII-1), D181 (GII-2), and IB80 (GVIII-1), have been detected over the years [11,12,13,14,15]. The GI-23 lineage has been circulating in chicken populations in Poland since its initial identification in 2015. As a result of its emergence and the subsequent introduction of a homologous vaccine, diagnosing the disease has become challenging, as it is difficult to distinguish between vaccine and field strains. The GI-23 lineage, which originated in the Middle East in the late 1990s and initially circulated locally, has expanded globally over the past 15 years. More specifically, it has been detected in Asia, Africa, Europe, and the Americas to date [16]. The factors driving this global spread remain unclear—whether they are due to human activity (such as poultry trade), the migration of wild birds, or specific biological traits of the virus that give it an advantage over other IBV lineages.

In the present study, we aimed to trace the evolution of the virus strain of the GI-23 IBV lineage, i.e., gammaCoV/Ck/Poland/G052/2016 (abbreviated as G052/16), during passages in chickens. We also sought to explore whether this lineage could adapt to Japanese quails (*Coturnix japonica*), as this species represents one of the smallest farmed poultry known to be susceptible to infections with both gamma- (including IBV) and deltacoronaviruses. Previous studies from Brazil and northern Italy have shown that quails can harbor IBV-like viruses and deltacoronaviruses [17,18,19]. In Poland, deltacoronavirus infections have also been detected in quails [20]. These findings confirm the previously suggested susceptibility of quails to become infected with both gamma- and deltacoronaviruses, providing an opportunity to use this poultry species as an animal model to investigate whether the GI-23 lineage can adapt to a new host and possibly identify the genetic factors involved in this adaptation. Moreover, such knowledge could also help explain the widespread global distribution of the GI-23 lineage.

To track the evolution of the IBV genome during passage in chickens and quails, we conducted an in vivo experiment with the GI-23 IBV lineage strain G052/16. Briefly, both species were intended to undergo ten passages of the G052/16 virus. Viral RNA was extracted from oropharyngeal swabs collected from infected birds at the specified time point of each passage and then sequenced using Next Generation Sequencing (NGS) to monitor the virus’s genetic evolution.

## 2. Results

### 2.1. Clinical Observations and Virus Load

No clinical signs of disease were observed in SPF chickens (2–3 weeks old) after infection with the virus strain. Only mild symptoms of diarrhea were noted on the 3 dpi in a few birds during different passages: CP-I, CP-V, and CP-VII. In the remaining passages, the birds were healthy. Viral RNA was detected in the oropharyngeal swabs from all passages with generally low viral loads (mean: 10^3^ copies/µL), except for passages CP-IV and CP-IX, where the viral load was higher (mean: 10^5^ copies/µL) (Table S1). In contrast, no clinical signs of disease were observed in quails, and viral RNA was detected at minimal levels only in the QP-I (Ct = 36.7, corresponding to approximately 2 virus copies/µL). In subsequent passages (QP-II–QP-V), no viral RNA was detected, and thus, further IBV passaging in quails within this experiment was discontinued. Instead, a follow-up experiment was conducted in which three quails were infected with a higher dose of the G052/2016 virus by administering an increased volume of viral material so that the birds were infected with 10^5,6^ EID_50_ and observed for a prolonged time of up to six days post-infection. On the third and sixth dpi, oropharyngeal and cloacal swabs were collected from each bird and analyzed for virus load in rRT-PCR. Again, no evidence of active virus replication was observed (no disease symptoms nor virus detection in oropharyngeal or cloacal swabs sampled), suggesting that quails are resistant to infection with the IBV GI-23 lineage.

### 2.2. NGS Sequencing and Variant Identification

Full-length nucleotide IBV sequences were obtained from oropharyngeal swabs throughout the ten passages in chickens. The average sequencing coverage ranged from 60,884 to 76,999, except for RNA extracted from CP-IV, CP-VIII, and CP-IX, where the coverage was lower (2378, 1980, and 2260, respectively) (Table S1). NGS analysis revealed a total of 62 SNVs, of which 30 led to amino acid changes, 28 were synonymous, and 1 was an insertion that introduced a stop codon in gene translation. Additionally, 3 SNVs were located in untranslated regions (1 in the 5′UTR and 2 in the 3′UTR) (Table S2). None of these variants persisted beyond seven passages. One synonymous SNV was detected in seven passages (C4779T and nsp3), and two SNVs were present in six passages (C10946T/A3488V/nsp8 and T20707A/Y130N/S). One non-synonymous variant was found in five passages (T7896G/F2471L/nsp4). The most prevalent changes were those that persisted for four or three passages, with 44 SNVs present in only one or two passages. The majority of variants appear at low frequencies (<15%), with consensus-level substitutions (i.e., >50% frequency) identified at 16 positions, including 8 non-synonymous mutations (Table 1). The CP-I showed mostly low-frequency variants, but by CP-IV, several variants had already reached consensus-level frequencies. Interestingly, the mutation with the highest frequency occurs in the S1 coding region in six passages, peaking at over 99% in CP-IX before dropping to 34% in the last passage (Y130N). The largest number of variants were observed in CP-IV, CP-VII, and CP-X (13 SNVs each), while CP-II, CP-V, CP-VI, and CP-IX contained 11–12 SNVs each. On the contrary, the CP-III contained the lowest number of SNVs (n = 6). One variant also appears in the inoculum and passage CP-II, but not in subsequent passages (Table S2).

### 2.3. Shannon Entropy and Viral Population Complexity

**Figure 1:**
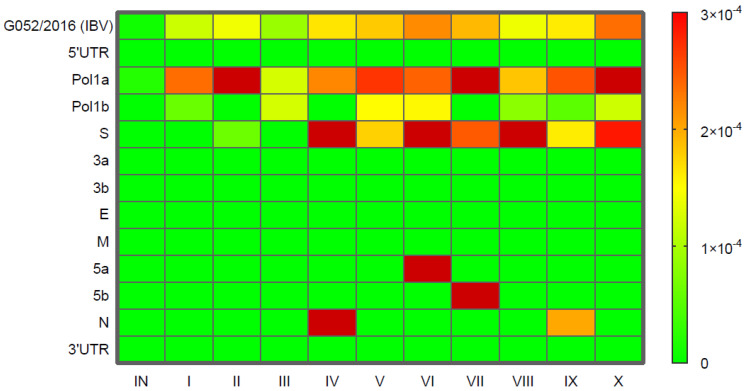
Heatmap of Shannon entropy reflecting the virus population complexity in the inoculum (IN) and each of ten passages (C−I – CP−X) at the level of individual genes and the whole genome. The color in the scale bar presents the value of Shannon entropy calculated from the frequencies of variants’ appearance. The heatmap was generated using GraphPad Prism 8 (Insight Partners, New York, NY, USA).

To assess the diversity of the viral populations, Shannon entropy was calculated separately for each IBV passage, including individual IBV genes as well as the entire genome. Entropy values were visualized using a heatmap (Figure 1). As indicated by the formula used (29), regions of the virus genome without variants resulted in Shannon entropy values of 0. Nevertheless, significant variations in entropy values throughout subsequent virus passages were also observed for the following genes: *pol1a*, *pol1b*, *S*, *5a*, *5b*, and *N*, and across the whole genome. The complexity of the IBV genome peaked in two passages, i.e., CP-VI and CP-X. The highest entropy values were observed in the *pol1a* gene (mean 2.29 × 10^−4^), with a peak at the CP-X (3.6 × 10^−4^). Moreover, high entropy was also noted in the *S* gene (mean 1.82 × 10^−4^), particularly in CP-IV, CP-VI, CP-VIII, and CP-X. For the *pol1b* and *N* genes, variations in entropy were also found, but these values were an order of magnitude lower (mean 6 × 10^−5^) compared to those in the *pol1a* and *S* genes. The highest entropy values in the *5a* and *5b* genes were recorded in CP-VI and CP-VII, respectively (Table S3).

### 2.4. Selection Pressure Analysis

The selection pressure profiles of the viral genes were analyzed by calculating the dN/dS ratios. Almost all genes exhibited dN/dS ratios less than 1, suggesting that these genes evolved under negative selection during passage. However, the dN/dS ratio for the *S* gene was slightly higher than 1, indicating potential positive selection. In total, eight codons were identified as being under positive selection, all detected using the FUBAR method with posterior probabilities > 0.9. These codons were located in *pol1a* (nsp2, nsp3, nsp4, nsp8) and *pol1b* (nsp12) genes (Table 1). The positively selected codons in *pol1a* included the following amino acid (aa) positions: 27 (L/F), 651 (R/K), 829 (K/R), 2373 (V/A), 2471 (F/L), 3488 (A/V), 3500 (L/F), and the aa position 20 (D/G) in *pol1b*. These variants appeared multiple times during the passages, with variants at aa positions 3500 (L/F) in *pol1a* and 20 (D/G) in *pol1b* appearing twice (in CP-I and CP-II or CP-IX and CP-X, respectively), and the variant at aa position 3488 (A/V) in *pol1a* appearing up to six times (CP-II, CP-IV, and CP-VI–CP-IX).

### 2.5. Gene S Evolution and Specific Variants

Although the *S* gene showed signs of evolution under positive selection, none of the codons altered in the *S* gene were identified as positively selected across all tested methods. Nevertheless, several non-synonymous variants were detected in the *S* gene. One variant, a substitution in the hypervariable region 2 (HVR2) at aa position 130 (Y/N), was present in six passages (CP-IV and CP-VI–CP-X). Another aa substitution (I/M) in the S2 coding region at position 937 appeared in three passages (CP-VII–CP-IX). A substitution at aa position 96 (F/L) in the S1 coding region was observed in two passages (CP-IV and CP-VI). The remaining variants, both synonymous and non-synonymous, were more transient, appearing in only one passage and then disappearing. Additionally, a synonymous SNV T22038C was found in the S1/S2 cleavage site (aa position 537) in the last passage (CP-X) (Table 1). SNVs detected in the *5a*, *5b*, and *N* genes were mostly spontaneous, appearing only in one passage each.

### 2.6. PSI-BLAST Analysis of Selected Variants

Variants identified as positively selected in pol1a and pol1b (n = 8) and also those found in the S protein were subjected to PSI-BLAST analysis (https://www.ncbi.nlm.nih.gov/BLAST/, accessed on 1 January 2025). Among the variants analyzed, the one at aa position 829 in nsp3 (K/R) was unique, as no identical substitutions were found in the database. The L/F substitution at aa position 3500 of nsp8 and the D/G substitution at aa position 20 of nsp12 were found in only 5 and 12 out of 500 viral strains, respectively. The remaining pol1a variants were detected in a comparable number of strains to those above. For the S protein, the substitution Y/N at aa position 130 was found in nearly 200 strains out of 249 analyzed, with the N variant found in only 8 strains; however, many strains had H or D substitutions at this position. Substitutions at aa position 937 (L) were observed in 20 out of 249 strains, while substitutions at position 452 (L, Y, and A) were rare. The results of the PSI-BLAST study show a rather low commonness of the identified changes. However, due to the very large diversity of IBVs, the results of our studies cannot be interpreted as evidence of evolutionary conservation of variants.

## 3. Discussion

In the current study, we investigated the genome evolution of the GI-23 lineage IBV during passages in chickens and explored the susceptibility of quails to infection with this virus lineage, as well as its potential adaptation for efficient replication in quails. Our results indicate that quails are not susceptible to infection with the IBV GI-13 lineage. The virus genome was detected only in the first passage on the 3 dpi of sampling, and the viral load was very low, likely due to the remains of the inoculum. No further virus was detected in subsequent passages. Previously, IB viruses of the GI-23 lineage have been identified in various wild bird species, although it remains unclear whether these birds serve as mechanical or biological vectors for the virus [21]. In this context, the Japanese quail appears to be a good animal model for experimental studies, as it represents a domesticated poultry species that may display migratory behavior, typical of wild species in their natural environment. Nevertheless, in our experiment, the virus did not actively replicate in quails, even though they are known to be susceptible to both gamma- and deltacoronaviruses [17,18,19,20]. However, it remains possible that some migratory wild bird species may be particularly susceptible to GI-23 IBV infection, potentially aiding its global spread. In contrast, substantial amounts of the virus were detected in all passages following the experimental infection of chickens. To date, many studies have confirmed that the IBV is well adapted to chickens and undergoes molecular changes during passage in their bodies, but no such detailed study of its evolution has been carried out, as planned in the present experiment. It is worth noting that the experiment was conducted on SPF birds, which did not exert immune pressure on the virus, thus minimizing the influence of immune response on virus evolution. The general view is that in the field, the immune response induced by previous vaccination is an additional factor influencing the emergence of new variants that thus evade immunity. The results obtained here clearly indicate that IBV is constantly changing during replication without the involvement of this factor. However, vaccination certainly accelerates this process, as the situation with SARS-CoV-2 has shown, when an increase in the percentage of genomic mutation rates was observed in the period after vaccination compared with the pre-vaccination sequences [22].

To quantify the genetic diversity of viral populations across subsequent passages, we calculated Shannon entropy and analyzed its changes over time. The highest average entropy measured for the whole IBV genome was found in the CP-X passage, while the lowest entropy values were observed in the CP-III. This suggests that viral variability did not stabilize even after passage in a perfectly adapted host, i.e., chicken, and in the absence of immune pressure, which is a factor that accelerates the genetic variability of most RNA viruses. Interestingly, this pattern contrasts with that seen in the study on the evolution of avian influenza virus (AIV), where the highest entropy is typically observed in early passages as the virus adapts to a new host. For instance, turkey-origin H9N2 AIV showed the highest entropy in the first two passages, which gradually decreased in subsequent passages [23]. Similar observations were made for the equine influenza virus H3N8 in embryonated chicken eggs [24]. However, unlike these studies, it seems that our experiment involved a very homogeneous inoculum, which contained only a single minority variant in the *pol1a* gene (H2839L). This inoculum underwent positive selection, resulting in a gradual increase in viral population diversity, starting from the CP-I. On the other hand, in both experiments with AIV, virus variants were detected with a frequency >2%, while in our experiment with a higher one, i.e., >5%. Hence, it is possible that numerous variants present in the 2–5% range were not identified, since a slightly higher threshold for variant detection was applied (>5%), specifically due to low coverage observed in particular passages (CP-IV, CP-VIII, and CP-IX). In this case, low-frequency variants detectable in samples with high coverage could be missed in these lower-coverage datasets, potentially introducing bias in the comparative analysis. As no significant correlation was found between Ct values and average sequencing coverage (*p* = 0.15, r = 0.45), and samples with similar Ct values yielded adequate coverage, the reduced coverage observed in certain passages is likely attributable to possible RNA degradation, which may have affected library preparation and downstream analysis.

Most of the newly emerging variants were minority variants, with a frequency below 15%, and primarily occurring within the *pol1a* gene. Starting from the CP-IV, consensus-level variants (adopted for this analysis at the level of frequency > 50%) appeared, predominantly affecting the *S* gene. The most genetically diverse genes were *pol1a* and *S*, though an interesting reciprocal trend in their diversity was observed: an increase in diversity within *pol1a* coincided with a decrease in diversity within the *S* gene (not confirmed by coevolution analysis). For the *5a* and *5b* genes, the incidental occurrence of single variants led to a substantial increase in Shannon entropy values in later passages (CP-VI and CP-VII, respectively) before these values declined rapidly to 0 in subsequent passages.

We observed that most mutations in the genome of IBV during 10 passages were transitions. Of all 63 identified mutations, only 7 were transversions, and the remaining 56 were transitions. Similar results were observed during passage of SARS-CoV-2, and they may result from a special feature of the RdRp of this virus [25]. The results obtained in this study confirm that this mutation type is also favored by the RdRp of IBV. These are not the only similarities to SARS-CoV-2. The highest mutation rates in SARS-CoV-2 within nsp3, S, N, and nsp12 were identified, regardless of vaccination status [22]. In our studies, most mutations were also identified within nsp3 and S. This pattern of changes may result from the function of these structures in viral fitness.

In the previous study published by Flageul et al. [26], the genetic diversity of IBV populations increased after the in vivo passage of the D388 strain of the GI-19 lineage in chickens, although only three passages were performed. Unlike our experiments, this study also compared the effect of post-vaccination heterologous immunity on virus population diversity. Notably, the inoculum used by Flageul et al. [26] was more heterogeneous, as it contained nine SNVs with a frequency of 8–26%. However, despite the homogeneity of our inoculum, as indicated by the presence of only a single low-frequency SNV (6.6%), it underwent positive selection and increased population diversity as early as the first passages. This observation suggests that virus evolution occurs regardless of the genetic diversity of the initial inoculum, but in the case of a homogeneous inoculum, newly formed variants take longer to reach the consensus level.

As referred to in the variant analysis, some variants that initially rose to high consensus-level frequencies (even >99%) were diminished or even eliminated in later passages (e.g., pol1a T7896G–F2471L, pol1b C17042T, and S 20707–Y130N). A possible explanation could be that these mutations were prevalent at higher frequencies only in a single bird, and the fact described above reflected the pooling of samples from three chickens.

Although Shannon entropy was highest in the *pol1a* and the *S* genes, the selection pressures acting on these genes were distinct. Specifically, the *pol1a* gene underwent negative selection, while the *S* gene was subjected to positive selection over time. Negative selection typically leads to the elimination of less-adapted variants, particularly those detrimentally affecting fundamental processes such as RNA replication and virus assembly [27]. Several codons in the *pol1a* gene, particularly within the nsp2, nsp3, nsp4, and nsp8 proteins, appeared to be under selective pressure, suggesting that mutations at these specific sites could provide advantages to certain virus variants. Interestingly, earlier studies on IBV attenuation have highlighted changes in the *pol1a* gene, especially in the nsp2, nsp3, or nsp4 regions, as key to the attenuation process [28,29,30].

To explore whether these mutations could be linked to virus attenuation, we compared the genome of the Var206 attenuated virus vaccine strain (TAbic^®^ IBVAR206, Phibro Animal Health Corporation, Teaneck, NJ, USA) with the IBV variants identified in our study. The vaccine strain showed 90.3% homology with the Polish G052/2016 strain used in our experiment. Three out of seven amino acids identified in our analysis as being under selective pressure were similarly altered in the vaccine strain. These particular changes occurred in nsp2, nsp3, and nsp4 (L27F, R651K, and V2373A, respectively), though the remaining four amino acids subjected to selective pressure were not present in the vaccine strain. While the non-synonymous changes observed in our study cannot be considered directly related to attenuation, they might reflect spontaneous mutations driven by positive selection. The functional significance of these identified positively selected sites remains unclear. On the other hand, structures with these sites are of paramount importance for the virus’s fitness. The first expressed nsp2 in the IBV genome cooperates in antagonizing the activity of host kinase, thus regulating the host immune response during virus replication. The largest nsp3 participates in polyprotein processing and has strong deubiquitinating and delsgylating activity, which has a regulatory effect on host cells to promote virus replication. The nsp4 induces membrane rearrangement, providing a site for the assembly and synthesis of viral RNA, and protects it from host immunity [31].

The *S* gene, which plays a critical role in host-virus interactions, underwent positive selection, which is expected as it influences viral entry and immune evasion [7]. Eight SNVs were identified within the *S* gene, five of which were non-synonymous, including one in the HVR2 region. Previous studies have shown that variations in the *S* gene can influence virus attenuation [30,32]. In addition, multiple changes within this structure were identified between viruses originating from different batches of the same GI-23 lineage vaccine [33]. A notable change, Y130N, was observed in the HVR2 region of the *S* gene in six of the passages. This position is near key residues involved in host cell affinity, particularly for tissues such as the respiratory, digestive, and urogenital tracts [34]. The virus used in our inoculum was propagated in SPF chicken embryos, which might explain the observed shift in viral tropism toward the respiratory system in later passages. Our analysis of the GenBank database revealed the Y/N/H/D substitution at the 130 positions; however, there was no information about which tissues the virus was identified from.

Nonetheless, the discussed results must be viewed in light of certain limitations. Specifically, an insufficient number of observations, such as the limited number of detected variants or the Shannon entropy metric yielding a single value per passage, hindered reliable statistical comparisons across all ten passages. As a result, a descriptive approach was adopted for data presentation. Future studies should aim to overcome these limitations by ensuring an adequate sample size and coverage to enable statistical analysis.

## 4. Materials and Methods

### 4.1. IBV Propagation in SPF Embryonated Eggs

The IBV GI-23 strain gammaCoV/Ck/Poland/G052/2016 (GenBank No: KY047602) with known genetic, virulence, and protectotype characteristics [35] was used to study its genetic evolution during passages in chickens. The virus was propagated in 9-day-old specific-pathogen-free (SPF) embryonated eggs (Valo BioMedia, Osterholz-Scharmbec, Germany) by inoculating the allantoic cavity. The inoculated SPF eggs were incubated for six days at 37 °C and candled daily for embryo mortality. At the end of the incubation period, allantoic fluids were collected. The viral titre was determined using the Reed and Muench method [36] and expressed as an embryo infectious dose (EID_50_). Virus material from the second passage in 9-day-old SPF embryonated eggs in a dose of 10^5^ EID_50_ was used to infect the first round of birds.

The same procedure was applied to materials from passages in chickens described below, with the exception that virus titers were not calculated. Instead, the collected fluids were used to extract RNA for further NGS analysis. Previous studies have shown that this approach does not alter the genetic population of the virus at the consensus level and ensures that 66% of the variants remain below the consensus [26].

### 4.2. Animals Used in Experiments

In the first experiment, a total of thirty SPF White Leghorn chickens aged between 2 and 3 weeks were used. These chickens were hatched from eggs purchased from an SPF egg producer (Valo-BioMedia GmbH, Germany) and reared to the appropriate age in the Animal Containment Level 3 (ACL-3) facility of our institute. For the second experiment, a total of thirty-one-week-old quails (*Coturnix japonica*) were purchased from certified breeders at the Department of Animal Genetics and Conservation, Institute of Animal Sciences, Warsaw University of Life Sciences (Poland), where the birds were subjected to ongoing welfare and health monitoring by veterinary staff. Throughout the experiment, the birds were housed in isolators under negative pressure (HM 1500, Montair Andersen BV, Kronenberg, The Netherlands).

### 4.3. Virus Passages in Birds

The animal experiments involving both poultry species followed the same procedural scheme. Three birds were inoculated with 10^5^ EID_50_/0.1 mL of the gammaCoV/Ck/Poland/G052/2016 virus by ocular and nasal routes. On the third day post-infection (dpi), oropharyngeal swabs were collected from each bird and placed in a transport medium (Copan, Brescia, Italy). The swabs from three birds were pooled, mixed thoroughly, and incubated for 15 min at room temperature before being divided into two parts. One part was used to infect three new birds with the same volume and inoculation routes. The other part was labelled as “Passage I” (P-I) and immediately frozen at −80 °C for further molecular studies. The process described for P-I was repeated for an additional nine passages, and the swabs from each subsequent passage were designated as “P-II through P-X” and frozen as before. For clarity, samples from chickens were marked with the prefix C, while samples from quails were marked with the prefix Q (e.g., CP-I or QP-I). For all passages except the inoculum (P0), samples were passaged once in SPF chicken embryos to optimize them for NGS as previously described [26]. Before inoculating the embryos, 200 µL of material was collected for RNA isolation and IBV load determination.

### 4.4. Assessment of Viral RNA Quantity in Chicken Swabs

Total RNA was extracted from the swabs using the RNeasy Mini Kit (Qiagen, Hilden, Germany) following the manufacturer’s instructions. The detection and quantification of viral load were performed using the QuantiTect Probe RT-PCR Kit (Qiagen, Germany), as described previously [37].

### 4.5. NGS of Viruses Passaged in Chickens

One hundred and eighty µL of filtered allantoic fluid was treated with 20 µL of TURBO DNAse (Invitrogen, Waltham, MA, USA) and 2 µL of RNase One (Promega, Madison, WI, USA) at 37 °C for 30 min to degrade nucleic acids not protected within viral capsids. RNA was extracted from 200 µL of the treated fluid using a combination of TRI (Sigma, Darmstadt, Germany) and the Direct-zol RNA MiniPrep (Zymo Research, Irvine, CA, USA) kit, with elution in 30 μL of DNase/RNase-free water. The prepared samples from different passages were sent simultaneously to a commercial sequencing service (Genomed Sp. z o.o., Warsaw, Poland). Upon receipt, the samples underwent further preparation for NGS (metagenomic approach). After DNase I treatment (New England Biolabs, Ipswich, MA, USA) at 37 °C for 30 min, both strand synthesis and DNA library preparation were performed using random primers and the NEBNext^®^ Ultra™ II RNA Library Preparation Kit for Illumina (Illumina Inc., San Diego, CA, USA). Sequencing was performed on the NovaSeq6000 platform (Illumina Inc., San Diego, CA, USA) to generate 150 bp paired-end reads.

### 4.6. NGS Data Analysis

Bioinformatics analysis, covering steps from quality assessment and filtering of raw data to generating consensus sequences, was conducted by an external company (Genomed Sp. z o.o., Warsaw, Poland). Briefly, adapter removal and quality filtering were performed with Cutadapt ver. 3.0., while de novo assembly, read mapping, and variant detection were carried out with CLC Genomic Workbench ver. 7.5. For further variant analysis, single nucleotide variants (SNVs) with a frequency of at least 5% in any virus passage were considered. Variant calling data were considered robust if at least 10 reads were present at all locations within the IBV genome (genome coverage ≥ 10).

To examine whether specific codon sites in individual genes of the virus were under positive or purifying selection pressure during IBV passaging in birds, we performed analyses using the Hy-Phy package (www.datamonkey.org, accessed on 24 March 2025). The ratio of non-synonymous (dN) to synonymous (dS) nucleotide substitutions per site (dN/dS) was calculated, along with selection pressure estimates using several methods (Fixed-Effects Likelihood-FEL, Single-Likelihood Ancestor Counting-SLAC, Fast Unconstrained Bayesian Approximation-FUBAR and Mixed Effects Model of Evolution-MEME) [38,39,40,41]. Codon positions confirmed as positively selected by at least one method were included in further analysis.

To assess the diversity of the viral populations, Shannon entropy was calculated for both individual IBV genes and the entire viral genome of each IBV passage (P-I–P-X) [42].

For the identified SNVs, Position-Specific Iterative Basic Local Alignment Search Tool (PSI-BLAST) analysis was performed to search for homologous sequences in the database using a score threshold of 500. A separate BLAST (v. 2.16.0+) search was conducted for the S protein specific to the GI-23 IBV lineage.

## 5. Conclusions

Our results have shown that quails were not susceptible to the IBV GI-13 lineage, with viral RNA detected only in the QP-I. In contrast, subsequent passaging of the IBV strain in chickens resulted in significant molecular changes, with the highest genetic complexity observed in the later passages (from the CP-I onward). This suggests that IBV variability does not stabilize in the absence of immune pressure. The *pol1a* and *S* genes showed the highest genetic diversity, with positive selection acting on the *S* gene and some codons within the *pol1a* gene. These findings suggest that the evolution of IBV is driven by host adaptation and highlight the potential for changes in virus tropism during passage.

## Figures and Tables

**Table 1:** Summary of selected viral variants in which the effect of selection pressure was identified and those that resulted in the change in the consensus sequence (frequency > 50% in bold). The exact passages in which the mutation was observed are given.

Gene	Structure	Nucleotide Variant	Amino Acid Change	Passage (Frequency %)	Selective Pressure
*pol1a*	nsp-2	C562T	L27F	CP-II (16.5) CP-III (5.1) **CP-V (67.0)**	yes
	nsp-3	G2435A	R651K	CP-VII (11.3) CP-VIII (15.7) CP-IX (11.8)	yes
		A2969G	K829R	CP-V (7.4) CP-VI (9.9) CP-VII (14.9) CP-X (10.4)	yes
		A3540G		CP-IV (14.1) **CP-VI (66.6)** CP-VIII (11.3) **CP-X (65.5)**	no
		C3871T	L1130F	CP-III (5.2) **CP-V (60.1)**	no
	nsp-4	C7601T	V2373A	CP-IV (6.3) CP-VIII (11.5) CP-IX (37.5) CP-X (28.1)	yes
		T7896G	F2471L	CP-II (7.7) CP-IV (14.5) CP-V (9.2) **CP-VI (82.3)** **CP-X (72.6)**	yes
	nsp-8	C10946T	A3488V	CP-II (5.5) **CP-IV (68)** CP-VI (42.7) CP-VII (11.3) CP-VIII (20.5) CP-IX (14.4)	yes
		C10981T	L3500F	CP-I (7.9) CP-III (14.1)	yes
*pol1b*	nsp-12	A12469G	D20G	CP-IX (15.4) CP-X (14)	yes
	nsp-14	C17042T		CP-VI (40.8) **CP-VIII (64.5)** **CP-IX (71.4)**	no
		C17776T	A1789T	**CP-V (68.3)**	no
	nsp-15	A18785T		CP-III (27.8) **CP-V (73.4)**	no
*S*	S1	C20429T	S37F	CP-II (5.7)	no
		C20607A	F96L	**CP-IV (67)** CP-VI (33.4)	no
	S1/HVR2	T20707A	Y130N	CP-IV (5.3) **CP-VI (51.5)** CP-VII (34.5) **CP-VIII (86.2)** **CP-IX (99.1)** CP-X (34.5)	no
	S1	C21183T		CP-IV (8.2)	no
		C21674T	S452L	**CP-V (69.2)**	no
	S1/S2	T22038C		CP-X (11.4)	no
	S2	-22703A	stop	CP-IV (5.7)	no
		A23130G	I937M	CP-VII (5.6) CP-VIII (37.5) CP-IX (20.4)	no

## Data Availability

The raw data supporting the conclusions of this article will be made available by the authors on request.

## Supplementary Materials

The following supporting information can be downloaded at: https://www.mdpi.com/article/10.3390/ijms26094200/s1.

## Abbreviations

The following abbreviations are used in this manuscript:

SNV	single nucleotide variant
QP-I/II	First/second virus passage in quails
CP-I/II	First/second virus passage in chickens
