# Supplementary material for: Genomic Alterations of the Infectious Bronchitis Virus (IBV) Strain of the GI-23 Lineage Induced by Passages in Chickens and Quails
Source: Int J Mol Sci. 2025 Apr 28;26(9):4200. doi: 10.3390/ijms26094200 (PMC12071609; doi:10.3390/ijms26094200)
Supplement: Supplementary file 1 [file ijms-26-04200-s001.zip › ijms-3577575-supplementary.pdf]

Table S1. Quantitation of viral load and coverage statistics for each sample sequenced.

Sample	Ct value	Virus load	Average coverage	SD
Inoculum	15.1	9,7x10^{6}	64076.13	22103.34
CP-I	33.0	1.03x10^{2}	76999.31	25645.15
CP-II	31.3	3.58x10^{2}	69157.85	23537.30
CP-III	27.1	4.75x10^{3}	65981.40	22249.06
CP-IV	20.6	2.95x10^{5}	2377.59	817.93
CP-V	29.4	9.62x10^{2}	75776.06	24705.49
CP-VI	30.2	5.85x10^{2}	71035.27	26288.60
CP-VII	30.2	5.85x10^{2}	60883.96	27693.38
CP-VIII	27.2	4.36x10^{3}	1979.08	830.22
CP-IX	22.4	1.03x10^{5}	2259.48	830.19
CP-X	27.6	4.13x10^{3}	72216.52	24424.09

SD – Standard Deviation

Table S2. Variants present in viral populations of the inoculum (IN) and each passage (CP-I – CP-X). Altered nucleotides (alt) with respect to the virus consensus sequence (ref) are given. Also reported is whether the altered nucleotides had an amino acid change with respect to the inoculum consensus sequence. The identified variants are presented along with the obtained frequency values (%).

Gene	Stucture	Nucleotide			Amino acid			IN	Passage (CP)									
		Position	Ref	Alt	Position	Ref	Alt		I	II	III	IV	V	VI	VII	VIII	IX	X
	5'UTR	89	G	A		syn												7.014516
pol1a	nsp2	535	C	T	18	L	A			6.281059								
		562	C	T	27	L	F			16.50625	5.078747		**67.01642**					
		1003	C	T	174	L	F					6.240822						
		1422	C	T	313	syn				5.15576								
	nsp3	1955	C	T	491	A	V		7.979878									
		2396	A	T	638	E	V								13.07993			
		2435	G	A	651	R	K								11.29827	15.71429	11.76471	
		2838	C	T	785	syn									27.17429			
		2969	A	G	829	K	R						7.377328	9.851186	14.88045			10.39206
		3120	A	G	879	syn							8.214486					
		3153	T	C	890	syn			11.61152									
		3540	A	G	1019	syn						14.13544		**66.56224**		11.34021		**65.51911**
		3871	C	T	1130	L	F				5.23792		**60.10319**					
		4779	C	T	1432	syn				38.1528	39.09253	6.62069		6.060606		21.2766	32.75862	15.86854
		4871	C	T	1463	T	I		7.587253									
		5184	C	T	1567	syn				34.17337								
		5252	C	T	1590	S	F							22.73647				
		5430	C	T	1649	syn									11.92512			
		6261	G	A	1926	syn							13.10091					
		6414	C	T	1977	syn			12.06683									
		6576	T	C	2031	syn											9.166667	
	np4	7419	C	T	2312	syn				6.872689								
		7601	C	T	2373	V	A					6.287425				11.49425	37.5	28.07435
		7896	T	G	2471	F	L			7.718183		14.46674	9.162783	**82.25824**				**72.57683**
		8135	C	T	2551	T	I								12.53892			
		8733	C	T	2750	syn							10.85441					
	nsp5	8889	C	T	2802	syn						6.462585						
		8998	C	T	2839	H	L	6.57		8.402746								
		9114	T	C	2877	syn						5.275779			13.21459			
	nsp6	10093	T	C	3204	syn												32.67008
		10265	C	A	3261	T	N						8.569848					
		10452	C	T	3323	syn				6.141345								47.7227
	nsp8	10946	C	T	3488	A	V			5.457127		**67.97386**		42.71255	11.27349	20.45455	14.43299	
		10981	C	T	3500	L	F		7.913952		14.08976							
		11108	C	T	3602	syn			12.51082									
		11289	T	C	3891	syn									11.11401			
	nsp10	12156	G	A		syn			20.79235									
		12233	A	G	3917	D	G		11.73964									
		12319	G	A	3946	F	A			6.703434								

between		12364	A	G		syn												11.1661
pol1b	nsp12	12469	A	G	20	D	G										15.38462	14.02226
		13591	C	T	394	syn					13.41829							
		14001	C	T	531	H	Y											25.04195
		15010	A	G	867	D	G		19.61778									
	nsp13	15680	C	T	1090	syn								23.17597				
	nsp14	17042	C	T	1544	syn								40.77079		**64.47368**	**71.42857**	
		17776	C	T	1789	A	T						**68.34217**					
	nsp15	18785	A	T	2125	syn					27.78151		**73.37851**					
S	S1	20429	C	T	37	S	F			5.669323								
		20607	C	A	96	F	L					**66.99545**		33.39223				
		20707	T	A	130	Y	N					5.346188		**51.47392**	34.54212	**86.2069**	**99.06542**	34.53831
		21183	C	T	288	syn						8.213945						
		21674	C	T	452	S	L						**69.19959**					
	S1/S2	22038	T	C	573	syn												11.35763
	S2	22703	-	A		STOP						5.71222						
		23130	A	G	937	I	M								5.635702	37.5	20.43011	
5a	5A	25468	G	T		syn								27.35849				
5b	5B	25697	G	A	11	G	R								12.36526			
N	N	26416	G	C	187	G	R										6.705539	
		26946	G	A	362	syn						29.35855						
	3'UTR	27457	C	T		syn									14.53362			
		27118	C	T		syn											5.803571	
**Number of variants in a passage (n)**								**1**	**9**	**12**	**6**	**13**	**11**	**11**	**13**	**8**	**11**	**13**
**Number of non-synonymous variants in a passage (n)**								**1**	**5**	**7**	**3**	**7**	**7**	**6**	**8**	**5**	**7**	**7**
**Number of synonymous variants in a passage (n)**								**0**	**4**	**5**	**3**	**6**	**4**	**5**	**5**	**3**	**4**	**6**

In bold - frequency values above > 50% level

Table S3. Shannon entropy calculation.

	IN	CP-I	CP-II	CP-III	CP-IV	CP-V	CP-VI	CP-VII	CP-VIII	CP-IX	CP-X	mean
**G052/2016**	8.78E-06	1.19E-04	1.42E-04	8.96E-05	1.65E-04	1.81E-04	2.18E-04	1.90E-04	1.41E-04	1.61E-04	2.35E-04	1.50E-04
**pol1a**	2.04E-05	2.36E-04	3.11E-04	1.25E-04	2.20E-04	2.69E-04	2.42E-04	3.02E-04	1.83E-04	2.51E-04	3.60E-04	2.29E-04
**pol1b**	0	6.21E-05	0	1.24E-04	0	1.51E-04	1.53E-04	0	8.16E-05	5.38E-05	1.21E-04	6.78E-05
**S**	0	0	6.24E-05	0	3.85E-04	1.77E-04	3.81E-04	2.47E-04	3.04E-04	1.60E-04	2.86E-04	1.82E-04
**3a**	0	0	0	0	0	0	0	0	0	0	0	0.00E+00
**3b**	0	0	0	0	0	0	0	0	0	0	0	0.00E+00
**E**	0	0	0	0	0	0	0	0	0	0	0	0.00E+00
**M**	0	0	0	0	0	0	0	0	0	0	0	0.00E+00
**5a**	0	0	0	0	0	0	2.98E-03	0	0	0	0	2.71E-04
**5b**	0	0	0	0	0	0	0	1.51E-03	0	0	0	1.37E-04
**N**	0	0	0	0	4.93E-04	0	0	0	0	2.00E-04	0	6.30E-05
**3'UTR**	0	0	0	0	0	0	0	0	0	0	0	0.00E+00
